# Role of Cardiac Rehabilitation in Improving Outcomes After Myocardial Infarction

**DOI:** 10.7759/cureus.50886

**Published:** 2023-12-21

**Authors:** Raghu Ram Shanmukh Nemani, Bala Sushritha Gade, Dedeepya Panchumarthi, Bhargava Venkata Sasidhar Reddy Bathula, Ganesh Pendli, Binay K Panjiyar

**Affiliations:** 1 Medicine, GSL Medical College, Rajahmundry, IND; 2 Medicine, Chalmeda Anand Rao Institute of Medical Sciences, Karimnagar, IND; 3 Medicine, PES Institue of Medical Sciences and Research, Kuppam, IND; 4 Research, Texas Tech University Health Sciences Center, Odessa, USA; 5 Internal Medicine, Harvard Medical School, Boston, USA; 6 Internal Medicine, California Institute of Behavioral Neurosciences & Psychology, Fairfield, USA

**Keywords:** reduced mortality and hospital readmission, improved quality of life (qol), positive outcome, cardiac rehabilitation (cr), post myocardial infarction (mi)

## Abstract

Myocardial infarction, an integral part of acute coronary syndrome (ACS), occurs due to atherosclerotic narrowing of the coronary (heart) blood vessels. Acute coronary syndrome, being one of the major cardiovascular diseases (CVDs), has led to a significant amount of mortality and morbidity, the majority of it due to MI. Over a long period following an MI, the physical, psychological, social, emotional, and occupational well-being are greatly impacted. Cardiac rehabilitation (CR) can address the above and help improve long-term well-being and overall quality of life. The benefits of CR include enhanced exercise capacity, risk factor reduction, improved quality of life (QOL), reduced mortality, and hospital readmissions.

We used a systematic literature review (SLR) approach in this article to provide a global overview of cutting-edge CR in the post-MI phase. We reviewed 45 articles from journals of good repute published between 2013 and December 1st, 2023, focusing on seven selected papers for in-depth analysis. The analysis was focused on factors such as the positive outcomes of CR and the effects of CR post-MI. There are only a few statistically significant studies in a few domains of CR benefits, namely decreased mortality, cardiac events, depression, depression-associated mortality, hospital readmissions, increased left ventricular ejection fraction (LVEF), left ventricular end-diastolic dimension (LVEDD), left ventricular end-systolic volume (LVESV), metabolic equivalent of task (MET), maximal oxygen consumption (VO2max), and the six-minute walk test (6MWT), and as a result, increased physical performance. Further research is needed to enhance the understanding of its mechanisms and statistically prove its effectiveness in all other domains. As CR continues to evolve, referral and participation in CR should be increased as it improves overall health and well-being.

## Introduction and background

**Figure 1:**
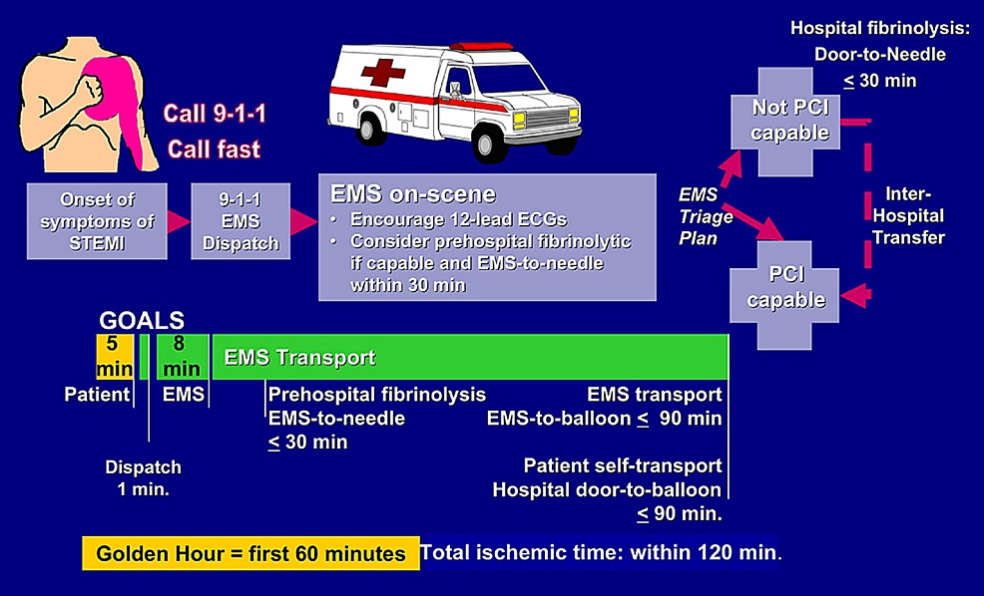
Management of STEMI STEMI: ST-segment elevated myocardial infarction Figure reused with permission from Antman, *Time Is Muscle Translation Into Practice*, Journal of the American College of Cardiology [2].

Myocardial infarction happens as a result of a partial or complete blockage of blood flow (coronary) to the heart muscle, leading to the ischemic death of the tissue supplied by that artery. Acute myocardial infarction (AMI) is classified into two types based on ECG findings: ST-segment elevated myocardial infarction (STEMI) and non-ST-segment elevated myocardial infarction (NSTEMI) [1]. STEMI occurs due to total occlusion of the blood flow [1], requiring the emergent treatment of percutaneous coronary intervention [PCI] or fibrinolytic therapy [2]. The primary aim of the healthcare system is to quickly identify and treat individuals experiencing STEMI, ensuring that the administration of fibrinolytic therapy begins within 30 minutes of medical contact (door-to-needle) or a PCI procedure starts within 90 minutes of medical contact (door-to-balloon), as demonstrated in Figure 1 [2]. An NSTEMI occurs due to subtotal occlusion of blood flow [1], which allows some flow of blood to the tissue.

As AMI is one of the most common cardiovascular diseases (CVDs), it continues to pose a significant health burden globally. Approximately 605,000 new instances of MI occur every year, along with 200,000 cases of recurring attacks. Within this total of 805,000 initial and repeated events, around 170,000 are believed to be asymptomatic [3]. The recent COVID-19 pandemic, still affecting populations due to its frequent emergence of new variants, significantly influenced cases of acute myocardial infarction (AMI). A few factors contributing to this were that, amidst the COVID-19 pandemic, timely interventions such as the door-to-balloon time faced delays [4], and the occurrence of arterial thromboembolic events following COVID-19 contributed to a rise in the frequency of AMI [5].

Over a long period following an MI, the physical, psychological, social, emotional, and occupational well-being were greatly impacted. Amid the high incidence and prevalence of AMI, there is an increasing need for effective interventions that address long-term health outcomes and quality of life. Cardiac rehabilitation (CR) is an emerging intervention targeting the above concerns. It includes various actions and approaches that empower patients to take control of their condition, prevent disease progression, and potentially reverse its course [6]. It is a critical component of cardiovascular care for patients with cardiovascular disease. Cardiac rehabilitation involves a structured program specific to each patient that typically lasts three to four weeks [6].

Cardiac rehabilitation is a cost-effective strategy with a holistic approach, encircling physical, emotional, and social aspects of patients' health-related quality of life (HRQOL) [7]. The primary aim of CR is to speed up the secondary prevention and improvement of patients’ quality of life (QOL). The core elements of CR comprise exercise training, lifestyle modification, and psychological intervention [6]. It extends beyond mere exercise routines to encompass comprehensive patient care, including drug therapy optimization, nutritional guidance, smoking cessation, stress management, and lifestyle improvement [8]. This comprehensive approach targets various aspects of patient health, ranging from physical fitness and cardiovascular risk factors to mental well-being.

Cardiac rehabilitation's goals include enhancing exercise tolerance, optimizing coronary risk factors (e.g., lipid profiles, blood pressure, glucose levels) [6], and addressing psychological aspects such as stress, anxiety, and depression [6, 9]. It brings about numerous positive outcomes. These encompass enhancements in exercise capacity, muscle strength, heart-related risk factors, and overall quality of life. Cardiac rehabilitation has been shown to improve various essential patient outcomes, including exercise capacity, control of cardiovascular risk factors, quality of life, hospital readmission rates, and decreased mortality rates [6]. The psychological aspects of CR address the lower quality of life that has been associated with mental illness after an MI attack [9]. Additionally, CR contributes to a reduction in mortality in patients with acute coronary syndrome (ACS) [8]. These advantages of CR extend to the elderly as well.

Individuals participating in CR witness a range of positive effects. Various studies have been done to prove this significance. The study by Carol et al. shows statistically significant improvement in levels of physical and mental quality of life in those who took part in CR [9]. The meta-analysis done by Ji et al., which included the group that underwent CR and the non-CR group, indicates a substantially lower hazard ratio, major adverse cardiac events (MACE), and recurrence rate of MI in the CR group, denoting a considerable benefit [8]. Heran et al. organized 47 different studies involving 10,794 patients who were randomly assigned to either CR or standard care. The results of their analysis reveal noticeable reductions in both overall mortality and cardiovascular mortality [6]. A retrospective cohort study by Kureshi et al. shows no statistically significant difference in general health status between patients who took part in CR and those who did not take part. However, the study states that participation in CR yields favorable survival benefits [10]. Furthermore, comprehensive evaluations of studies consistently emphasize the decreased mortality risk linked to engaging in CR. This has resulted in designating referral to CR as an important component of cardiac care [10].

This article was previously posted to the medRxiv preprint server on 20th October 2023.

## Review

Systematic literature search methods and study selection

Focusing on the outcomes of CR in post-MI patients, we conducted a thorough search for relevant publications by using databases like PubMed, Medline, Cochrane Library, MDPI, and Google Scholar. We performed reference checking and forward-citation searching of all primary studies and review articles to identify additional studies that satisfied our inclusion criteria. The preferred reporting items for systematic review and meta-analyses (PRISMA) 2020 standards were developed to make reporting more thorough and transparent. These were followed throughout the entire investigation.

Search strategy

The population, intervention/condition, control/comparison, and outcome (PICO) criteria were utilized to conduct a thorough literature review. The search was conducted using relevant keywords, such as cardiac rehabilitation, post-myocardial infarction, and positive outcomes. The medical subject heading (MeSH) approach for PubMed and other databases (Table 1), was employed to develop a comprehensive search strategy.

**Table 1:** Search strategy, search engines, and the search results displayed

	Database	Search Strategy	Search Results
1.	PubMed	(Cardiac Rehabilitation[Title/Abstract]) OR (Post Myocardial Infarction[Title/Abstract])	178
		((Positive Outcome[Title/Abstract]) AND (Cardiac Rehabilitation[Title/Abstract])) OR (Myocardial Infarction[Title/Abstract])	2074
		(Cardiac Rehabilitation[MeSH Terms]) AND (Post Myocardial Infarction[MeSH Terms])	3
		All Fields:- (Cardiac Rehabilitation) OR (Outcomes in Post Myocardial Infarction)	562
2.	Cochrane Library	Title Abstract Keyword:- "cardiac rehabilitation" OR "post-myocardial infarction"	20
3.	MDPI	Abstract:- Cardiac Rehabilitation OR Outcomes in Post Myocardial Infarction	219
4.	Google Scholar	Role of Cardiac Rehabilitation in Improving Outcomes after Myocardial Infarction	125

We combined the articles in an EndNote (Clarivate, London, UK) for duplicate removal. The records were initially reviewed based on titles and abstracts, and irrelevant articles were excluded based on our inclusion and exclusion criteria (Table 2), followed by a review of full-text articles.

**Table 2:** Inclusion and exclusion criteria

	Inclusion Criteria	Exclusion Criteria
1.	Human studies	Animal studies
2.	From 2013 to 2023	Only methodological studies explaining programming details
3.	English Text	Non-English texts
4.	Gender: All	
5.	Age: > 19 years of age	Age:<=19 years
6.	Free papers	Papers that needed to be purchased
7.		Studies involving clinical data other than cardiovascular diseases

Results

After searching through three selected databases, PubMed, PMC, Medline, Cochrane Library, and MDPI, we extracted a total of 3181 articles after combining 2817 in PubMed with 20 Cochrane Library articles, 219 MDPI articles, and 125 Google Scholar articles. EndNote was used to remove 497 duplicate articles, leaving 2683 items in total for screening. A total of 2535 articles were removed after the title and abstract screening; 103 articles were not retrieved; and 38 out of 45 articles were disqualified for the reasons mentioned in the PRISMA flow chart after being assessed for eligibility. Following the screening, seven papers were included in our final systematic review. Table 3 provides a detailed description of each.

**Table 3:** Details of the results of the selected papers CR: Cardiac rehabilitation

Author and year of publication	Study design	Database used	Conclusion
Bellmann et al., 2020 [6]	Comprehensive Review	Google Scholar	The three main measures of CR and the effectiveness of CR for acute or chronic cardiovascular disease.
Francis et al., 2019 [7]	Meta-analysis	Google Scholar	The improvement of health-related quality of life (HRQOL) was demonstrated in those who took part in comprehensive CR.
Ji et al., 2019 [8]	Meta-analysis	PubMed	CR's association with reductions in recurrence of MI, and cardiac mortality
Carol et al., 2018 [9]	Cohort study	MDPI	CR's association with higher levels of physical and mental quality of life and lower levels of depression
Faraz et al., 2016 [10]	Retrospective cohort study	Google Scholar	Participation in CR conferred survival benefits even though not statistically significant
Tessler et al., 2019 [11]	Comprehensive review	Google Scholar	The components and phases of CR.
Powell et al., 2018 [12]	Systematic review and meta-analysis	PubMed	Study of difference between CR participants and non-participants in all-cause mortality, cardiovascular mortality, and hospital admissions

**Figure 2:**
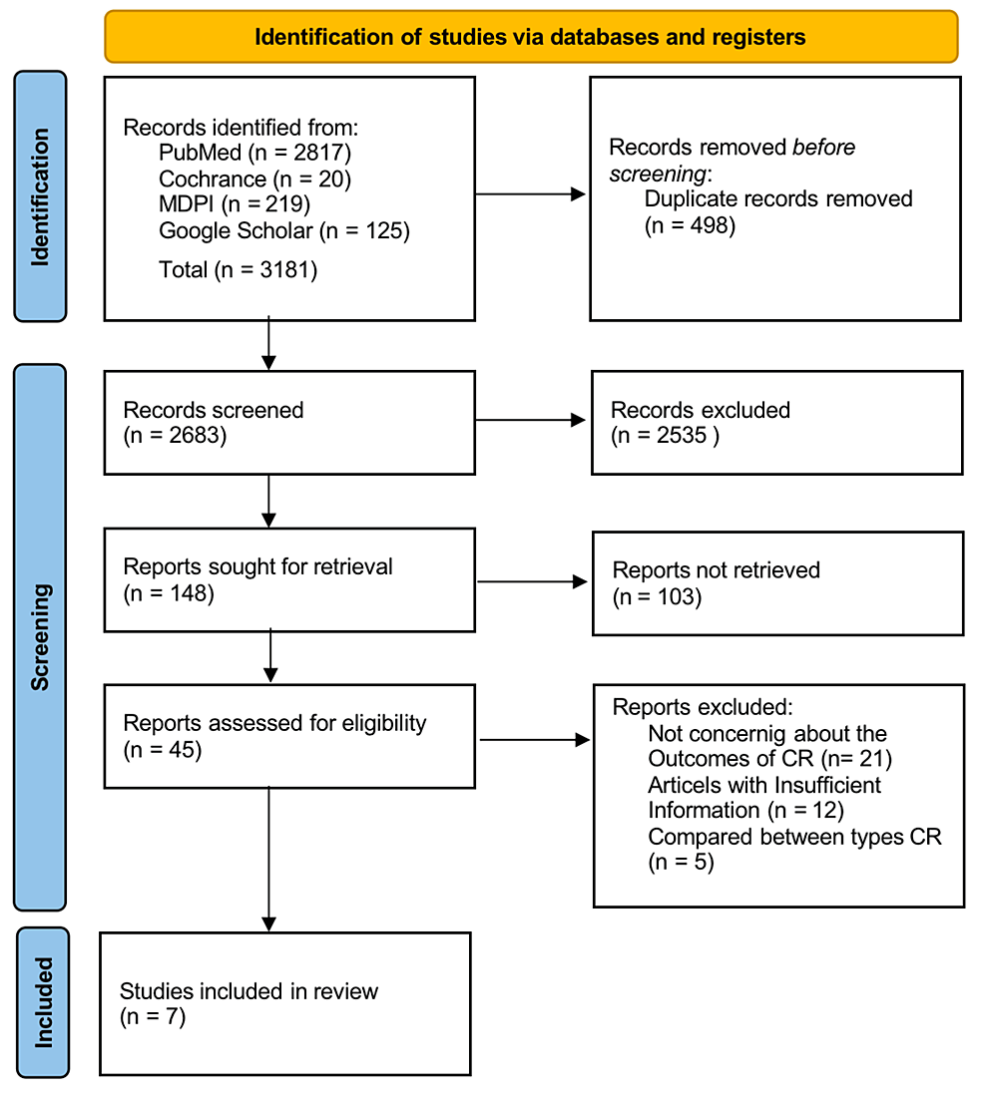
Flow diagram of PRISMA illustrating the search strategy and study selection process for the systematic review PRISMA: Preferred reporting items for systematic reviews and meta-analyses

A PRISMA flow chart [13] representing the selection of articles is shown in Figure 2.

Quality appraisal

To ensure the reliability of our chosen papers, we utilized various quality assessment tools. We employed the PRISMA checklist and Cochrane bias tool assessment for randomized clinical trials for systematic reviews and meta-analyses. Non-randomized clinical trials were evaluated using the Newcastle-Ottawa tool scale. We assessed the quality of qualitative studies (Table 4) using the critical appraisal skills program (CASP) checklist. To avoid any confusion in the classification, we utilized the scale for the assessment of narrative review articles (SANRA) to evaluate the article's quality.

**Table 4:** Quality appraisal tools used PRISMA: Preferred reporting items for systematic reviews and meta-analyses, SANRA: Scale for the assessment of non-systematic review articles

Quality appraisal tools	Types of studies
Cochrane bias tool assessment	Randomized control trials
Newcastle-Ottawa tool	Non-RCT and observational studies
PRISMA checklist	Systematic reviews
SANRA checklist	Any other study without a clear method section

Discussions

**Figure 3:**
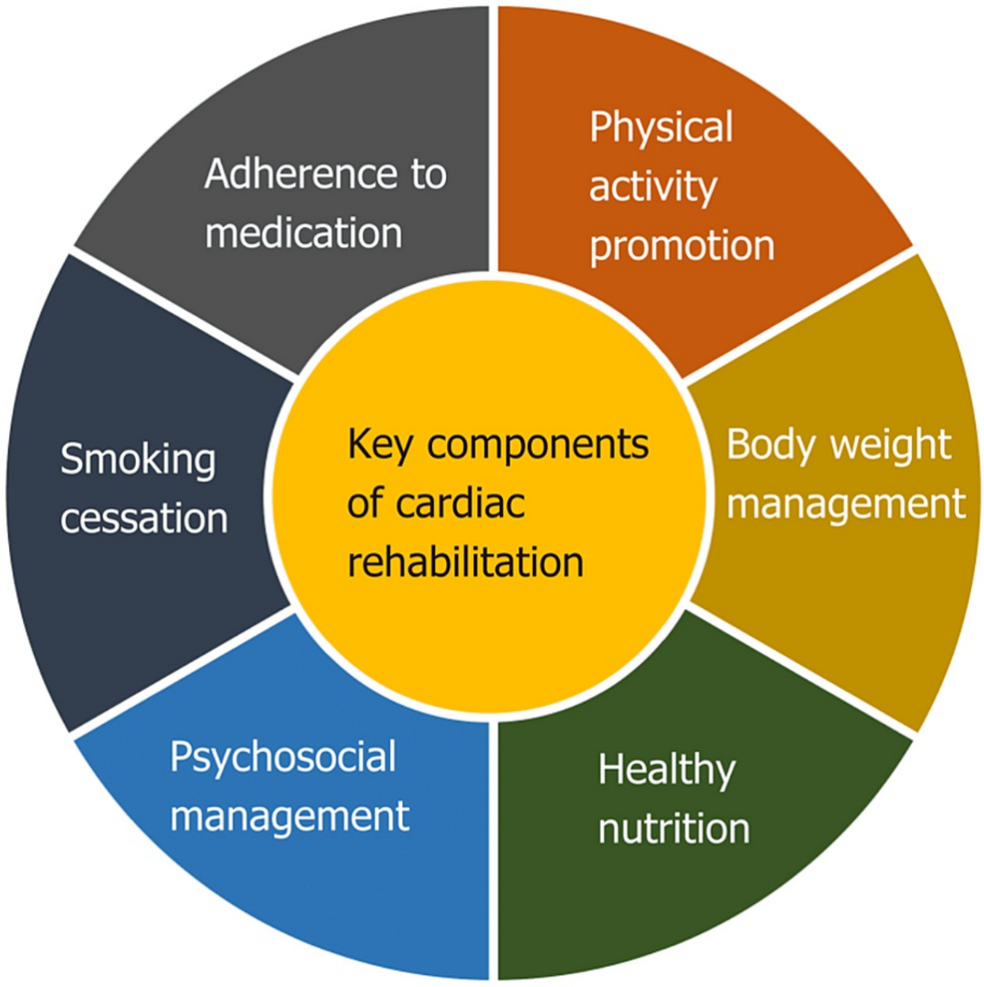
Key components of cardiac rehabilitation Figure reused from *Remotely Monitored Telerehabilitation for Cardiac Patients: A Review of the Current Situation *by Batalik et al.[14], an open-access article with a Creative Commons Attribution NonCommercial (CC BY-NC 4.0) license.

Cardiac rehabilitation consists of three phases [11]. The first is the clinical phase, which starts after the heart event or completion of the intervention by assessing the patient's physical abilities. It includes guiding the patient through non-strenuous exercises in the bed or bedside. The second phase is outpatient rehab, which begins post-stability and is cleared by cardiology. It has a custom exercise plan tailored to specific patients based on assessments focusing on identifying limitations in physical function and activities. The third phase is post-rehab, which enhances flexibility, strength, and aerobic fitness. Patients are encouraged to maintain an active lifestyle. Regular outpatient check-ups are advised to monitor cardiovascular health and medication regimens. The components of CR include patient assessment, nutritional counseling, weight management, blood pressure management, lipid management, diabetes management, tobacco cessation, psychosocial management, physical activity counseling, and exercise training. Figure 3 [14] shows the components of CR.

The precise mechanisms through which CR contributes to improving the prognosis for individuals with AMI remain not fully understood [8]. However, CR offers numerous physiological advantages due to its emphasis on physical activity. Participating in exercise training has been proven to enhance maximal oxygen uptake (VO2max) and the flow of blood to the heart muscle [11]. It also leads to decreased smoking, body weight, serum lipids, and blood pressure [11]. As a part of CR, exercise training has been shown to enhance the endothelium-dependent dilation of coronary arteries in patients with ischemic heart disease [15]. Cardiac rehabilitation has shown various direct benefits for the cardiovascular system. These encompass improvements in the supply of oxygen to the heart muscle, the functioning of the endothelial layer, the balance of the autonomic nervous system, factors related to blood clotting, markers of inflammation, and the growth of collaterals for blood flow in the coronary arteries [8]. These effects of CR are proof that it has a positive impact on the patient.

A meta-analysis of RCTs by Yanijao et al. shows a statistically significant improvement in the control group regarding left ventricular ejection fraction (LVEF), left ventricular end-diastolic dimension (LVEDD), and left ventricular end-systolic volume (LVESV)[ 16]. A study by Agnieszka et al. shows that the exercise stress test time and the metabolic equivalent of task (MET), the maximal oxygen consumption (VO2max), and the six-minute walk test (6MWT) score increased significantly (p = 0.0001) [17]. This leads us to the conclusion that CR significantly improves physical performance in patients after MI.

Cardiac rehabilitation has consistently demonstrated significant reductions in overall cardiovascular mortality and hospital admission rates. Studies conducted by Heran et al. and others have indicated a decrease in cardiovascular mortality and improved exercise capacity among patients participating in exercise-based CR [6]. A study by Powell et al. shows a reduction in hospital admission following CR that was statistically significant [12]. A study conducted by Shah et al. demonstrates that engaging in mild to moderate CR was linked to a lower likelihood of experiencing post-infarction angina and irregular heartbeats [18]. The above studies suggest that CR plays a crucial role in enhancing patient survival and reducing the likelihood of recurring cardiovascular events.

One of the noticeable advantages of CR is its positive impact on patients' quality of life (QOL). Several studies have evaluated the effect of CR on QOL primarily using the SF 36 QOL questionnaire, and their results consistently indicate that CR leads to significant improvements in various domains of QOL [19,20]. For instance, the study by Carol et al. shows statistically significant improvement in levels of physical and mental QOL [9]. Another study shows that ACS patients who took part in CR exhibited significant increases in QOL scores across all domains [21]. This improvement in QOL is attributed to the comprehensive nature of CR, which addresses not only physical health but also psychological and social aspects.

The psychological aspect of CR consists of psychosocial evaluation, in which the interviewer uses standardized tools to find out if someone is struggling emotionally, especially with feelings of sadness or anxiety [9]. The ways to help include group learning, talking to someone about it, learning skills to manage stress, and support for making healthier lifestyle choices. It's suggested to involve family, and if needed, the person might be referred to mental health experts [9]. Three studies have demonstrated a statistically significant reduction in depression and anxiety among participants in CR [9,22,23]. This suggests that a decrease in depression is linked to a corresponding decrease in mortality associated with depressive conditions. Furthermore, a study has also provided statistically significant evidence supporting the reduction in mortality associated with decreased depression [23].

Yoga-based CR leads to enhanced self-assessed well-being and a return to activities as they were before the heart attack, following an AMI. However, this study by Prabhakaran et al. does not have enough statistical strength to demonstrate a distinction in major adverse cardiovascular events (MACE). In cases where traditional CR is inaccessible or not preferred by individuals, yoga-based CR could present itself as a potential alternative [24].

**Figure 4:**
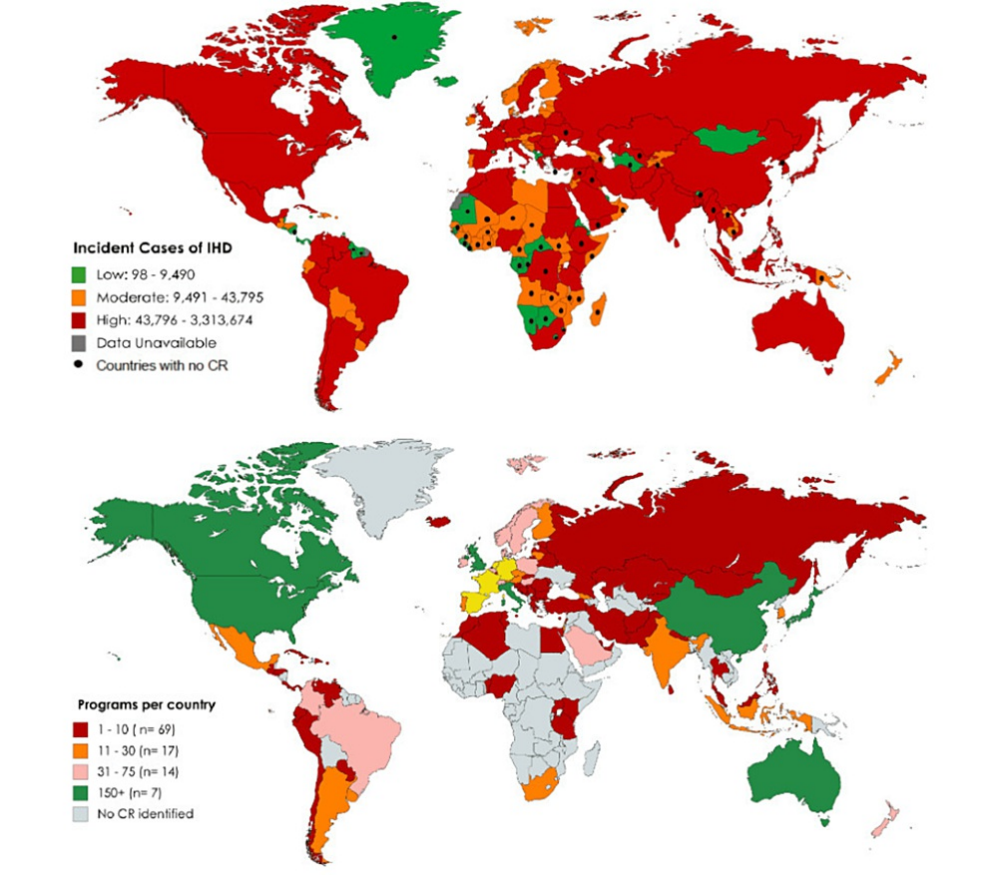
Global incidence of ischemic heart disease and availability of CR CR: Cardiac rehabilitation Figure reused from *Cardiac Rehabilitation Availability and Density Around the Globe* by Turk-Adawi et al. [25], an open-access article with a Creative Commons Attribution NonCommercial (CC BY-NC 4.0) license.

Due to these numerous favorable effects, it is essential to promote greater participation and referral of CR in numerous countries globally. This is particularly important since many nations experiencing a high incidence of ischemic heart disease possess a limited quantity of CR programs, as evident from the data presented in Figure 4 [25].

Despite the substantial evidence supporting the benefits of CR, several limitations should be acknowledged. Variability in the design of CR programs, differences in patient populations, and varying lengths of follow-up periods can introduce heterogeneity in study outcomes. Moreover, some studies have failed to show statistically significant differences in certain outcomes. Future research should aim to standardize CR interventions and implement rigorous methodologies to enhance the robustness of findings.

Limitations

Our literature review has limitations. We limited our analysis to English articles published within the last 10 years, specifically targeting those at least 19 years old. We also only used free articles, and our study was limited to English papers on CR post-MI. More research is needed for specific conclusions.

## Conclusions

Cardiac rehabilitation is an integral component of cardiovascular care, recommended by clinical guidelines to improve patient outcomes and their quality of life. By addressing both physical and psychological aspects, CR contributes to the improvement in various domains of QOL, including reduction of cardiovascular mortality, depression, depression-associated mortality, post-infarction angina, irregular heartbeats, hospital admissions, and adverse events. Statistically significant evidence only in a few domains underlines the value of CR in patient well-being and long-term cardiovascular health. Continued research and efforts to promote the widespread adoption of CR programs are crucial to ensuring better outcomes for patients with MI.
